# Pneumonia detection with QCSA network on chest X-ray

**DOI:** 10.1038/s41598-023-35922-x

**Published:** 2023-06-03

**Authors:** Sukhendra Singh, Manoj Kumar, Abhay Kumar, Birendra Kumar Verma, S. Shitharth

**Affiliations:** 1 JSS Academy of Technical Education, Noida, India; 2 National Institute of Technology Patna, Patna, India; 3 Kebri Dehar University, Kebri Dehar, Ethiopia

**Keywords:** Computational models, Image processing, Machine learning

## Abstract

Worldwide, pneumonia is the leading cause of infant mortality. Experienced radiologists use chest X-rays to diagnose pneumonia and other respiratory diseases. The diagnostic procedure's complexity causes radiologists to disagree with the decision. Early diagnosis is the only feasible strategy for mitigating the disease's impact on the patent. Computer-aided diagnostics improve the accuracy of diagnosis. Recent studies established that Quaternion neural networks classify and predict better than real-valued neural networks, especially when dealing with multi-dimensional or multi-channel input. The attention mechanism has been derived from the human brain's visual and cognitive ability in which it focuses on some portion of the image and ignores the rest portion of the image. The attention mechanism maximizes the usage of the image's relevant aspects, hence boosting classification accuracy. In the current work, we propose a QCSA network (Quaternion Channel-Spatial Attention Network) by combining the spatial and channel attention mechanism with Quaternion residual network to classify chest X-Ray images for Pneumonia detection. We used a Kaggle X-ray dataset. The suggested architecture achieved 94.53% accuracy and 0.89 AUC. We have also shown that performance improves by integrating the attention mechanism in QCNN. Our results indicate that our approach to detecting pneumonia is promising.

## Introduction

**Figure 1:**
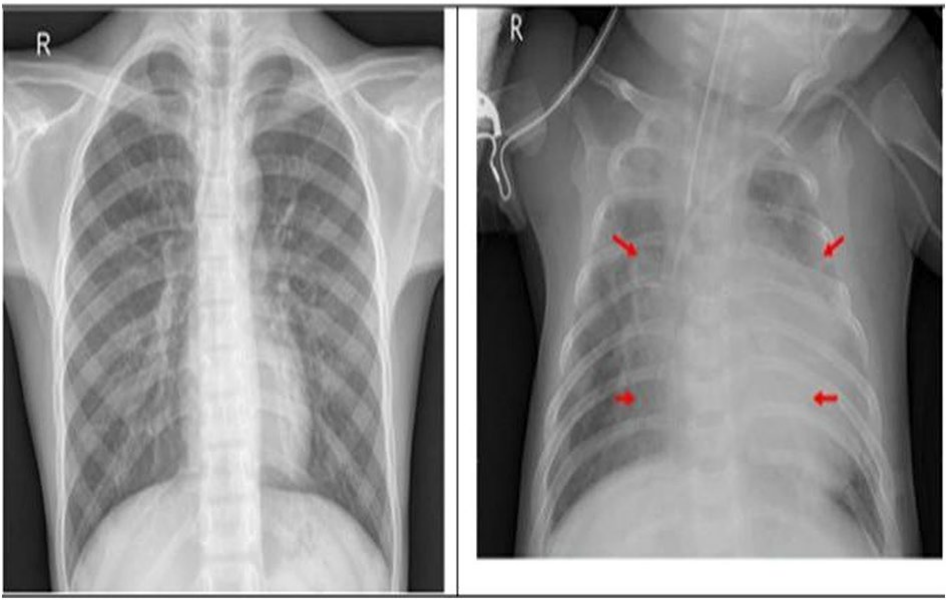
A sample of CXR scan (normal and pneumonia).

Pneumonia is an infection of the lungs that may be due to bacteria, viruses, or fungi. This infection inflames the air sacs and fluid-filled lungs (pleural effusion). Pneumonia is a leading cause of infant mortality and worldwide death. Overcrowding, pollution, and an unhygienic environment lead to pneumonia in underdeveloped and developing nations with few medical resources. Our work will benefit millions of people worldwide by providing a promising approach to accurately and efficiently detect pneumonia from chest X-ray images, which facilitates early diagnosis and treatment, and ultimately improve patient outcomes. Early detection and treatment are key to averting a fatal condition. X-rays, CT, MRI, and CT are used to diagnose lung disorders, among which X-Ray is most commonly used for the diagnosis of pneumonia. The proposed architecture will help radiologists to accurately analyze X-rays, CT, MRI, and CT, which could lead to diagnosing other respiratory diseases, bone fractures, and tumors. The advantages of QCSA lie in its ability to effectively capture the complex spatial and channel-wise correlations in chest X-ray images, which is crucial for accurately detecting pneumonia. Figure 1 depicts the CXRs of a person with pneumonia and a healthy individual. The white dots on the CXR on the right indicate the presence of pneumonia. Pneumonia CXR testing is subjective to radiologists' experience, and hence computer-aided assistance for detection and diagnosis is required for an accurate result.

Deep neural networks have exhibited exceptional image classification potential^1^. However, most current research on image classification architectures is based on real-valued data. The study^2^ has argued that the real-valued CNN cannot properly encode the relationship between multi-image channels. Quaternion number systems were utilized to address this issue, which are generalizations of complex number systems and have remarkable properties that can be exploited to create more robust designs. In our study, we have built quaternion convolution neural networks (QCNN), which are CNN extensions. The QCNN^3^ can extract the most representative features from multiple-dimensional input objects. This is because the orientation and spatial position of color image channels within the input images are encoded correctly in QCNN.

The attention mechanism^4,5^ has attracted significant interest in computer vision systems for object recognition and scene interpretation in the past few years. By focusing only on the relevant parts of an object, the human visual system enables people to discriminate objects quickly when it is viewed. The capability of the human brain has inspired the use of attention mechanisms in deep neural networks^6^. The attention mechanism was utilized more frequently in activities linked to natural language processing^7^. It has also lately been used for image classification tasks^8^ to produce cutting-edge outcomes. Channel and spatial attention processes^9^ are the two typical types of attention mechanisms applied in computer vision tasks.

Recently, researchers^10–13^ have experimented with the quaternion extension of CNN and produced outperforming results compared to real-valued CNN. In this experiment, the channel and spatial attention modules of a quaternion residual quaternion network were employed to improve the performance of predicting Pneumonia from CXR images. The capability of quaternions to adequately describe spatial transformations and evaluate multi-channel data makes them an intriguing candidate for computer vision applications.

The novelty of the proposed work is that we have incorporated a spatial attention layer in between the layers of Quaternion convolutional neural network. This enables the network to learn important regions from the chest X-ray images while attending to complex spatial features, thereby improving the accuracy of pneumonia detection. Our analysis of the feature map and the attention map shows that the QCSA network is able to effectively learn features from important regions of the chest X-ray images which leads to better performance of the proposed framework to detect pneumonia.

### Major contributions

The following is our contribution to this experiment.

1. We have first built residual quaternion architecture and evaluated the performance of Pneumonia detection on the CXR dataset.
2. We then incorporated spatial and channel attention modules in the architecture in (i) and kept all hyper-parameter values the same for both architectures. We then evaluated the performance of this attention-augmented architecture.
3. We then compared the performance of both the architecture to compute the influence of incorporating spatial and channel attention modules.

The remainder of this work is structured as follows. The background necessary for the proposed work and recent research results in issue areas connected to the proposed work is presented in “Background and similar works” section. “Materials and methods” section outlined the properties of the utilized dataset and recommended design. “Experimental analysis” section presents hardware infrastructure, performance metrics, and experimentation details and “Analysis of result” section describes a discussion of results. The conclusion and future scope of the proposed work is given in “Conclusion and future work” section.

## Background and similar works

Here, we give the necessary background ideas for the suggested design as well as a comparative study of the findings of other recent investigations related to the same problem area.

### Quaternion convolution neural network (QCNN)

QCNN^14^ is an extension of the real CNN model. Quaternion is a four-dimensional vector space having a basis of 1, i, j, and k. One of these orthogonal subspaces is a scalar subspace of one dimension, whereas the other is a pure subspace of three dimensions. Quaternion neural networks are a more recent form of neural network that uses quaternion-valued inputs, activation, and parameters (QNNs). Quaternions are numbers with one real component and three imaginary components. Each of its three imaginary components may encode a color component of an RGB image, making them appropriate for image processing. Numerous proposed models outperform their real-valued equivalents in tasks such as image processing and speech recognition^15,16^ in recent years. Moreover, quaternion-valued networks benefit from parameter sharing as a result of the interactions of the Hamilton product^17^, resulting in models that require fewer parameters and less storage space and are hence smaller. These benefits can be provided to representations by substituting quaternionic layers for conventional (real-valued) layers, hence lowering their size without a perceptible decrease in performance.

Inputs and layers of a QNN have quaternion values as opposed to real values. Although the work on quaternion representations for deep learning is in its infancy, few papers analyzing their value have been published. Deep quaternion networks have been used specifically for classification^18,19^ and segmentation^20^. According to their research, quaternions offer superior results for a variety of tasks while necessitating fewer parameters. QCNNs were developed in order to correctly display color images in the quaternion domain. They found that their QCNN models for color image classification^21^ and denoising outperformed traditional CNNs. The authors of^22^ studied the influence of the Hamilton product on the grayscale-only reconstruction of color images. To reconstruct a unique grayscale image, a quaternion convolutional encoder-decoder architecture is created in^12^. In contrast to standard convolutional encoder-decoder networks, their method can efficiently learn to reconstruct an image's colors from its grayscale representation. They conclude that quaternion-valued systems are unfettered by internal and global dependencies, making them suited for applications involving image recognition. Quaternion Recurrent Neural Networks (QRNNs) are proposed by the same authors^23^ for sequential tasks such as speech recognition. Their quaternion-based recurrent designs beat non-quaternion-based alternatives despite having two to three times fewer parameters.

**Figure 2:**
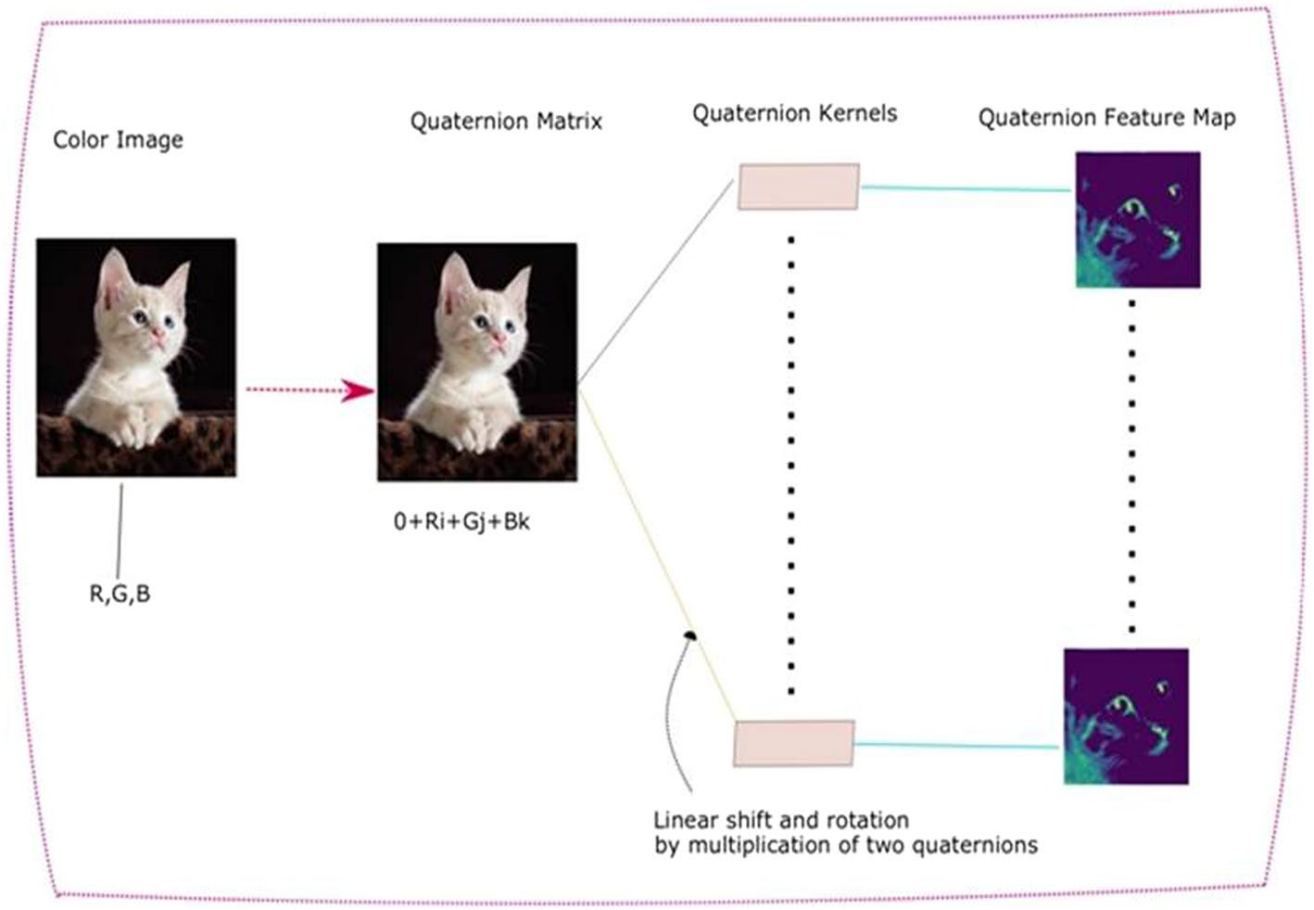
Building Block of a generic Quaternion CNN.

Figure 2, shows the building blocks, which show the customization of conventional CNN into quaternion CNN.

### Algebra of quaternion numbers

This section describes identities and properties^24^, followed by quaternion numbers.

Following Eq. (1) is the notation for a quaternion Q.

1 $$Q = r + xi + yj + zk$$

Furthermore, the Imaginary components of Quaternion can be expressed by Eq. (2).

2 $$i^{2} = j^{2} = k^{2} = ijk = - 1$$

As seen by the following Eq. (3), the product of two quaternions violates the commutative property.

3 $$ij = k = - jijk = - kj = iki = - ik = j$$

Also, in the quaternion domain, r represents the scalar component, x, y, and z represent the imaginary component in xi + yj + zk, and v represents the vector component. It has been represented by Eq. (4).

4 $$Q = (r,v)$$

The conjugate of Q is denoted by Eq. (5).

5 $$Q^{*} = {\text{(r}} - {\text{xi}} - {\text{yj}} - {\text{zk )}}$$

The magnitude of Q is shown by ||Q|| is described by Eq. (6).

6 $$||Q|| = \sqrt {r^{2} + x^{2} + y^{2} + z^{2} }$$

The inverse Q^−1^ of a quaternion Q is defined by the expression as given in Eq. (7).

7 $$Q^{ - 1} = \frac{{Q^{*} }}{{||{\text{Q}}||^{2} }}$$

Just like a complex number, a quaternion number can also be represented as in Eq. (8).

8 $$Q = \rho e^{\theta s} = \rho (cos\theta + ssin\theta )$$

ρ =|Q|, θ is a real quantity and s is a pure imaginary quaternion of unit length.

Rotate a three-dimensional vector Q by an angle along a rotation axis w to obtain a new vector p. This rotation may be shown in Eqs. (9) and (10).

9 $$\widehat{{\text{Q}}} = q_{1} {\text{i}} + q_{2} {\text{j}} + q_{3} {\text{k}}\quad {\text{and}}\quad \widehat{{\text{p}}} = {\text{p}}_{1} {\text{i }} + {\text{p}}_{2} {\text{j}} + {\text{p}}_{3}$$

$\widehat{{\text{p}}} = { }\widehat{{\text{w }}} \cdot \widehat{{\text{Q}}} \cdot { }\overline{{\widehat{{\text{w }}}}} \,{\text{where}}\,{\hat{\text{p}}}\,{\text{and}}\,{\hat{\text{Q}}}$ are pure Quaternion with the real component being zero

10 $$\hat{w} = cos\frac{\theta }{2} + sin\frac{\theta }{2}(w_{1} + w_{2} + w_{3} )$$

The Quaternion convolution method employs scaling and rotation between the Q and Q_N_ input filters.

Here, w is a quaternion filter of size F, and Q is a quaternion matrix of size N. Then, as in Eq. (11), the quaternion operation can be written as.

S = N − F + 1 and T = N − F + 1

11 $$\left\{ {\begin{array}{*{20}l} {\hat{Q}\hat{w} = \left[ {\widehat{{f_{{kk^{\prime } }} }}} \right] \in H^{(S) \times (T)} } \hfill \\ {ff_{kk}^{\prime } = \mathop \sum \limits_{i = 1}^{M} \mathop \sum \limits_{j = 1}^{M} \frac{1}{{s_{ij} }}w_{ij} q(k + i)\left( {k^{\prime } + j} \right)\overline{{w_{ij} }} } \hfill \\ {w_{\prime } = s_{\prime } \left( {cos\frac{{\theta_{\prime } }}{2} + \mu sin\frac{{\theta_{\prime } }}{2}} \right)} \hfill \\ \end{array} } \right.$$

Here, s stands for the scaling component is the axis of unit length, and fluctuates between—and. Due to the Hamiltonian product, as indicated in Eq. (11), A QNN can represent the local and global dependence inside the multi-channel input's features.

#### Hamiltonian product

In QCNN, the Hamilton product is utilized in place of the conventional real-valued dot product to carry out the following transformations between two quaternions,

Q_1_ = r_1_ + x_1_i + y_1_j + z_1_k and W_1_ = r_2_ + x_2_i + y_2_j + z_2_k, here Q_1_ and W_1_ are two quaternions.

 ⊗ operator is used to represent the Hamiltonian product of two quaternions Q_1_ and W_1,_ and it is defined as Eq. (12).

12 $${\text{Q}}_{{1}} \otimes {\text{W}}_{{1}} = ({\text{r}}_{{1}} {\text{r}}_{{2}} - {\text{ x}}_{{1}} {\text{x}}_{{2}} - {\text{ y}}_{{1}} {\text{y}}_{{2}} - {\text{ z}}_{{1}} {\text{z}}_{{2}} ) + ({\text{r}}_{{1}} {\text{x}}_{{2}} + {\text{ x}}_{{1}} {\text{r}}_{{2}} + {\text{ y}}_{{1}} {\text{z}}_{{2}} - {\text{ z}}_{{1}} {\text{y}}_{{2}} ){\text{i}} + {\text{(r}}_{{1}} {\text{y}}_{{2}} - {\text{ x}}_{{1}} {\text{z}}_{{2}} + {\text{ y}}_{{1}} {\text{r}}_{{2}} + {\text{ z}}_{{1}} {\text{x}}_{{2}} {\text{)j}} + {\text{(r}}_{{1}} {\text{z}}_{{2}} + {\text{ x}}_{{1}} {\text{y}}_{{2}} - {\text{y}}_{{1}} {\text{x}}_{{2}} + {\text{z}}_{{1}} {\text{r}}_{{2}} {\text{)k}}$$

The Hamilton product enables QNN to discover latent interactions inside the Quaternion's properties. During the Hamilton product in a QNN, the quaternion-weight components are shared over many quaternion-input sections, hence forming connections between the elements. In a real-valued neural network, the multiple weights necessary to encode latent relations within a feature are evaluated at the same level as learning global dependencies between different features, while the quaternion weight w encodes these interconnections within a unique quaternion Q_out_ during the Hamilton product.

### Attention mechanism

Image attention involves finding a target region as the eye rapidly scans the image. When smaller activation values are combined by the associated feature map, a substantial quantity of feature map information is discarded.; therefore, combining spatial and channel attention in the quaternion-residual network produces superior results. Second, regions of interest are highlighted as opposed to feature maps. When channel attention reduces the information in individual feature maps, spatial attention can highlight numerous significant regions of each feature map by employing the attention mask of a different branch. In the last phase, the output feature maps of two attention processes are concatenated. These characteristics of interest are amplified in fused feature maps, while redundant features are deleted. To collect the most accurate target data while reducing unnecessary data, this target region is weighted (distributed). Soft attention^25,26^ is the most popular since it is differentiable and trains CNN models from start to end. Most soft attention models employ an attention template to locate distinctive aspects for aligning the weights of discrete sequences or image segments. Hard attention, as opposed to soft attention, is a stochastic, non-differentiable procedure that analyzes distinct regions as opposed to the image's primary characteristics. The attention network for image classification can determine an image's attention spectrum's weight of the arithmetic mean of attention. The method can gather image-based attention like natural language processing.

Because it collects features from data, a deep neural network can classify images pixel-wise. The attention mechanism^27^ mimics human vision and helps identify significant characteristics quickly and precisely. CNN process all image information and details in all convolution layers. Multiple convolution layers and global average pooling in the last layer average the image's characteristics and attributes. This network's last affine fully connected layer determines image classification. Background and other non-essential information have a greater impact on categorization results as image size decreases. Large quantities of data plus a neural network that learns not to emit background information prevent outcomes from being inaccurate.

One way to generate one image from two or more convolution layers is to branch the output of one layer. We set sigmoid, the convolution output activation function, to work a value between zero and one for each pixel. Sigmoid keeps input values within the range of 0 to 1. The result of the convolution function multiplies the initial output. The two further layers assess the output's quantity. Near-zero values are unimportant. This configuration discards most sigmoid values approaching zero from the downstream recognition process. Configuring a neural network to estimate the area of focus using the result is the most common way to use attention for image classification.

Literature^27^ has produced two visual-system-inspired attention strategies. The first is a top-down method that iteratively selects the correct region from a scene record pool. The bottom-up approach, however, highlights the most critical visual path places. Top-down iteration is slower than bottom-up. The bottom-up technique selects the most relevant regions from incoming data progressively, although sequential processes increase errors with depth.

The attention mechanism is a prominent study topic for many reasons. Any model's attention mechanism outperforms baseline techniques. Second, using backpropagation, the attention model can be trained with a base recurrent neural network. The transformer model's^28^ induction was widely used in image processing, video processing, and recommendation systems, improving the attention model and avoiding the parallelized issue in recurrent neural networks.

Classification neural networks model data as a numeric vector of low-level features with the same weights against their capabilities. The attention model assigned variables to features based on their relevance. The attention model computes the weight distribution based on the input features and assigns greater values to features with high rank.

The attention mechanism has three layers: alignment, attention weight, and context vector. The attention layer calculates the alignment score between the encoded vector h = {h_1_, h_2_,….. h_n_) and a vector v. As stated in Eqs. (13) and (14), the SoftMax computes the probability distribution α_1_ by normalizing over all n elements of h where i = 1, 2,…n.

13 $$\propto_{i} = \frac{{exp^{{\left( {h_{i}^{\prime } v} \right)}} }}{{\mathop \sum \nolimits_{j = 1}^{n} exp^{{\left( {h_{i}^{\prime } v} \right)}} }}$$

14 $$O = \mathop \sum \limits_{i = 1}^{n} \propto_{i} {\text{h}}_{{\text{i}}}$$

From the equations above, hi provides vector v with vital information. The attention mechanism output O is a weighted sum of the encoded vector hi.

In the proposed work, we have combined channel attention and spatial attention mechanism in quaternion residual networks.

#### Channel attention

Using the inter-channel relationship between features, a channel attention^26,29,30^ map is created. As each channel of a feature map is seen as a feature detector, the channel focuses on global features. It reduces the spatial dimension of the input feature map in order to appropriately compute channel attention. The channel attention method generates a sigmoid-activated one-dimensional (1-D) tensor for specified feature maps. In a few channel axes of feature maps, it is anticipated that some activation values of the 1-D tensor will be larger than the corresponding feature maps of interest, but others will be smaller so as to prevent the repetition of feature maps. We generate two spatial context descriptors, F_Avg_^c^ and F_max_^c^, which stand for average-pooled features and max-pooled features, respectively.

#### Spatial attention

On the basis of the interstitial interaction between features, a spatial attention map is generated. In contrast to channel attention, which focuses on a channel's location, the spatial attention module emphasizes the location of an important feature. To compute spatial attention, we first apply the average-pooling and maximum-pooling processes along the channel axis, then concatenate the results to provide a useful feature descriptor. The concatenated feature descriptor is used in conjunction with a convolution layer to build a spatial attention map that encodes where to highlight or suppress.

**Figure 3:**
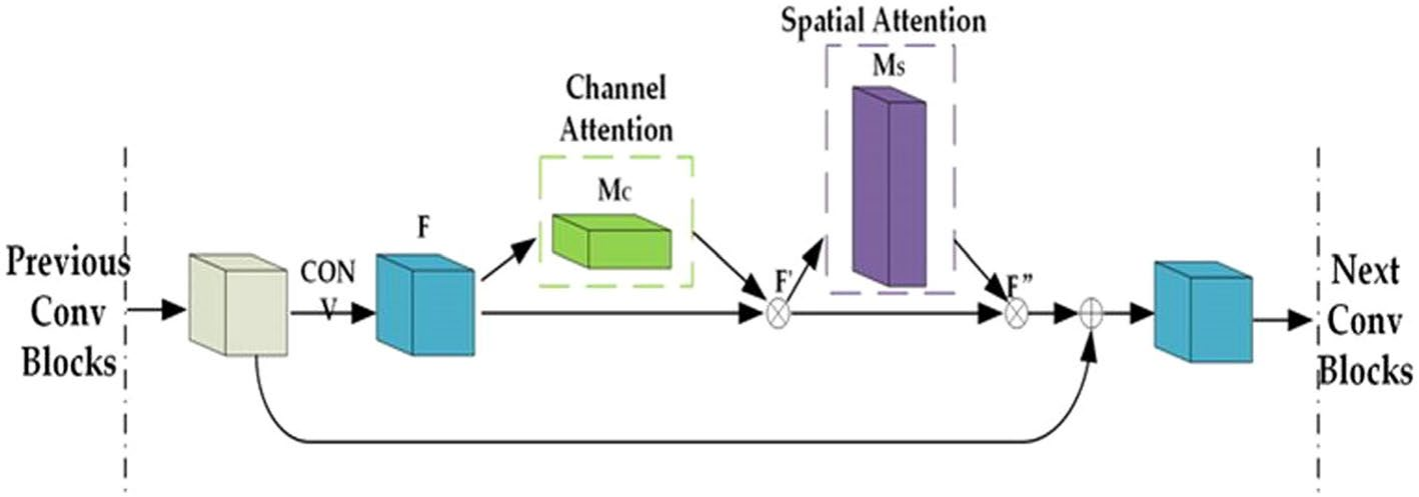
Augmentation of Channel and spatial attention modules in building blocks of CNN.

Figure 3 shows how we placed channel attention and spatial attention blocks inside the building block of QCNN. These spatial and channel blocks were compatible with quaternion inputs. Adding channel and spatial attention blocks do not increase learnable parameters and hence does not give computational cost.

### Comparison of recent related studies

Pneumonia detection via CXR has been an unresolved issue for many years, with the lack of publicly available data constituting the primary limitation. Extensive research has been conducted on traditional machine learning algorithms, which require domain expertise for feature extraction. Deep learning models produced a variety of architectures, such as VGGNet^31^, ResNet^32^, Inception ResNet^33^, etc., which were used with transfer learning techniques^34^ employing pretrained weights. Recent strategies for detecting pneumonia are split into three categories: (1) those in which researchers have prioritized region of interest extraction, (2) methods emphasizing feature extraction, followed by typical machine learning models or an ensemble of models with average performance, (3) a deep learning architecture based on transfer learning. Table 1 described the recently studied literature.

**Table 1:** Literature summary of recently related studies.

Study's reference	Approach	Findings	Limitations
^35^	It utilized a weighted ensemble of GoogLeNet, ResNet-18, and DenseNet-121. The weights assigned to the classifiers were determined by combining the hyperbolic tangent function with the accuracy, recall, f1-score, and area under the curve (AUC) assessment measures	The proposed approach delivered an accuracy rate of 98.81%, a sensitivity rate of 98.80%, a precision rate of 98.82%, an F1-score of 98.79 on the Kermany dataset	The computational complexity associated with training the model was reasonably significant. Image quality can be improved using contrast enhancement or other preprocessing techniques. Before classifying lung pictures, we may additionally segment them to better feature extraction in CNN models
^36^	The CNN model included 21 layers of depth-separable convolutions. A depth-separable convolution can isolate the most efficient section of a standard convolution while rejecting the rest	It achieved an accuracy of 94.87%, a sensitivity of 96.70, a specificity of 91.90, and an AUC of 98.80	When the magnitude of Gaussian noise rose, its performance dropped substantially
^37^	This effort involved the categorization of three classes. It utilized VGG19 and transfer learning techniques using weights that were previously trained	It achieved 97.11% accuracy, medium precision, and 97% recall	The employed dataset is modest in size
^38^	Proposed an ensemble of ChexNet and VGG19, and retrieved features were used for ensemble classification using the random forest approach	Prediction accuracy of 98.93% and AUC 0.99	The model was overfitted by the ensembled architecture's large number of trainable parameters
^39^	This work used Kaggle CXR images to create the extreme learning machine (ELM). They applied PCA for feature extraction with contrast-enhanced by contrast, restricted adaptive histogram equalization on CXR images	the ELM model with CLAHE and hybrid CNN-PCA beat all other models	A large dataset is required for better results
^40^	They suggested a multitask deep learning technique capable of recognizing COVID-19 patients and segmenting COVID-19 lesions from chest CT images simultaneously. Segmentation, classification, and reconstruction are performed concurrently on distinct data sets. Reconstruction, segmentation, and classification were conducted, respectively, using a shared encoder, two decoders, and a multilayer perceptron	With a dice coefficient more than 0.88 for segmentation and a region under the ROC curve greater than 97% for classification, the reported results demonstrate the technique's very promising performance	If patient information other than CT images could have been used, the findings may have been improved
^41^	CheXNet architecture has been presented to identify Pneumonia from CXR samples. CheXNet is a 121-layer CNN that was trained on the largest publicly accessible CXR dataset, which includes over one hundred thousand frontal-view CXR images with fourteen diseases	On the F1 measure, they determined that CheXNet beats the average	Training time larger

## Material and methods

### Dataset

The dataset^42^ (https://www.kaggle.com/datasets/paultimothymooney/chest-xray-pneumonia) is organized into the train, test, and validation directory, with a subdirectory for each image type (**P**neumonia/**N**ormal) within each directory. There are 5,856 CXR images in JPEG format, split into two categories (P/N). The CXR images of one- to five-year-old infants at the Guangzhou Women and Children's Medical Center were chosen retrospectively from cohorts. CXRs were frequently taken as part of the patient's therapy. Before the images could be used to train an AI system, two expert physicians reviewed them. A third expert evaluated the assessment set more thoroughly to account for any potential grading problems. The training set comprised 5136 images; however, the test set only has 700. Table 2 displays the datasets for each classification.

**Table 2:** Class wise distribution of thedataset.

Class	No of images
Pneumonia (P)	4273
Normal (N)	1583

Table 3 demonstrates that 75% of the dataset has been allocated to the training set, 80% to the test set, and 20% to the validation set.

**Table 3:** Train, test, and validation dataset partitioning.

	# of images	# of images from P class	# of images from N class
Training data	4392	3205	1187
Validation data	292	226	66
Test data	1172	842	330

### Proposed framework

The proposed method comprises image preprocessing with an image enhancement technique and image resizing, dataset imbalance handling, augmentation of training images, the transformation of input images into the quaternion domain, training on a Quaternion residual network with spatial and channel attention modules, and evaluation of Pneumonia classification with the proposed model. Figure 6 depicts our suggested design, which augments the structure of quaternion residual network architecture with channel and spatial attention modules.

#### Data preprocessing

In preparation for image normalization, the photos are converted into an array and sorted by 255. It allows the scale of an image to be specified between 0.0 and 1.0. It helps each image by removing abnormalities caused by shadows and illumination.

#### Image enhancement

Image quality affects the performance, and we performed it also to maintain uniformity in the entire dataset input images.

#### Data augmentation

By applying various types of transformation on input images, challenges of smaller dataset size is rectified.

#### Dataset balancing

It is done to maintain a balance between the input data size of all dataset classes.

#### Training of proposed architecture

The preprocessed dataset is projected in quaternion space and trained on the QCSA network.

#### Evaluation of performance

Trained model is then tested on unseen images to evaluate its performance.

**Figure 4:**
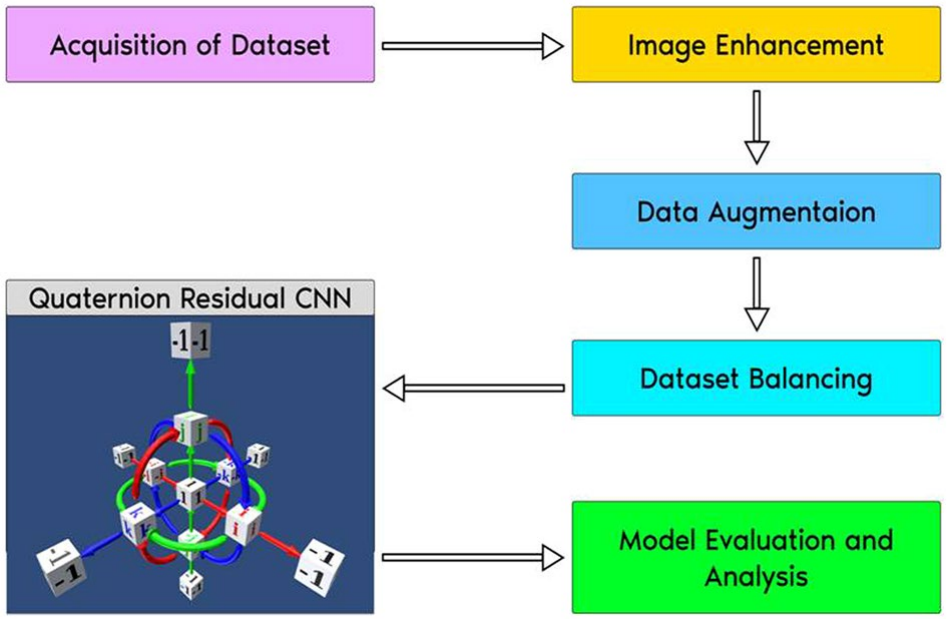
Workflow in the experiment.

Figure 4 diagrammatically shows the steps carried out in our experiment, which include preprocessing steps on the selected dataset, design of proposed architecture, training of model on the preprocessed dataset, followed by testing of evaluation of the performance of proposed architecture.

**Figure 5:**
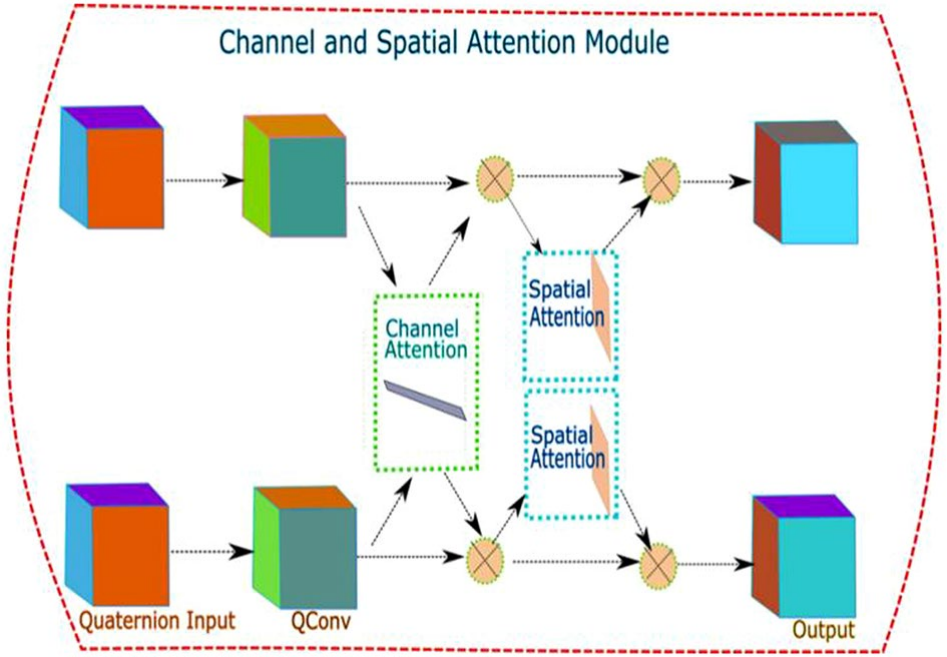
Building block of a QCSA network.

Spatial and channel attention modules focus only on the crucial part of the input and extract features from them only. Figure 5 shows the relative positioning of spatial and channel attention blocks in the proposed architecture.

**Figure 6:**
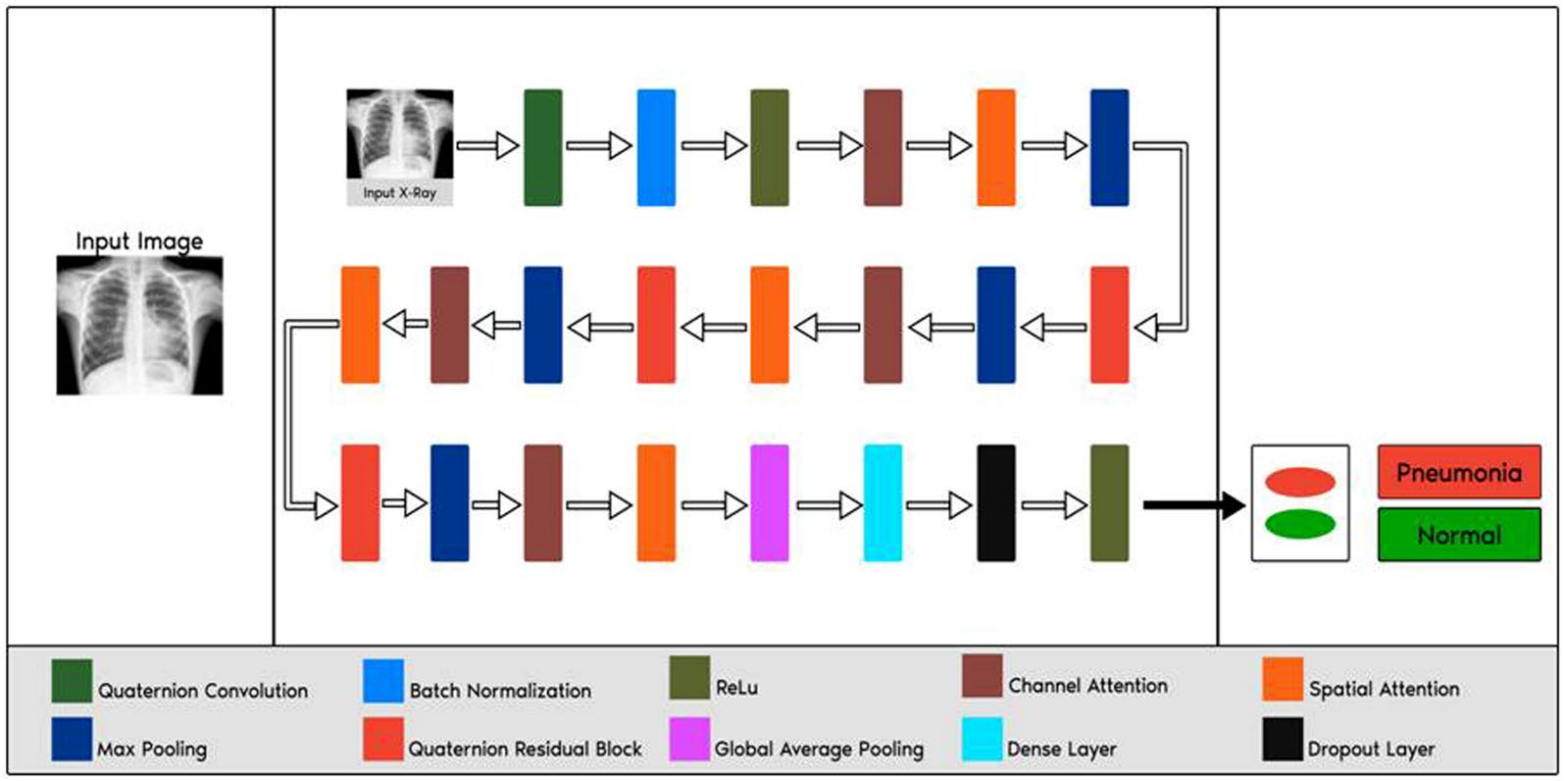
Proposed architecture design.

Figure 6 displays the design of the proposed architecture, which shows the detailed structure of the proposed model. In this, we have employed four quaternion residual blocks with attention blocks.

## Experimental analysis

### Implementation details and hyper-parameter settings

To showcase our proposed architecture, we experimented with one of the most commonly downloaded datasets for testing on Kaggle, a benchmark dataset of CXR images. Utilizing these research and datasets for binary categorization. Python 3.7, Anaconda/3, and CUDA/10 are installed on a Windows server with an i5 CPU, 2 GB GPU, and 8 GB RAM. In addition to the aforementioned parameters, the Python libraries Tensorflow-Keras, OpenCV, matplotlib, os, math, and NumPy are employed. As shown in Table 4, we have trained the system for 40 epochs using hyperparameters.

**Table 4:** Hyperparameter setting used in the experiment.

Hyper parameter	Value
Optimizer	Adam
Loss function	Binary cross-entropy
Batch size	16
Number of epochs	40
Learning rate	0.001
Decay	1e−6
Image resize	50 × 50
Stride	(1,1)

### Performance metrics

Accuracy, precision, recall (or sensitivity), the F1 score, and specificity are used to evaluate the performance of the proposed system with respect to the binary classification problem at hand. True Positive (TP), True Negative (TN), False Positive (FP), and False Negative (FN) must be defined prior to defining these concepts. Assume that the two classes in a problem of binary classification are positive and negative. TP refers to the classification of a sample as positive. FP refers to a sample that has been incorrectly categorized as positive when it actually belongs to the negative class. In a similar manner, TN refers to a sample that has been correctly categorized as a member of the negative class. FN refers to a sample that is classed as negative despite belonging to the positive class.

#### Accuracy

It is the proportion of correctly classified samples to the total number of samples.

$$Accuracy = \frac{TP + TN}{{TP + TN + FP + FN}}$$

#### Precision

The proportion of properly recognized Positive samples to the total number of Positive samples determines precision (either correctly or incorrectly). Precision is the degree to which a model correctly identifies a sample as positive.

$$Precision = \frac{TP}{{TP + FP}}$$

#### Recall

The Recall is calculated as the proportion of correctly recognized Positive samples compared to the total number of Positive samples. Recall measures the model's capacity to recognize Positive samples. As recall grows, an increasing number of positive samples are detected.

$$Recall(sensitivity) = \frac{TP}{{TP + FN}}$$

#### F1-score

The F1-score combines precision and recall as a measurement. Typically, it is stated as a harmonic mean of precision and recall.

$$F1Score = \frac{2*Precsion*Recall}{{Precsion + Recall}}$$

#### Sensitivity

It is a test's capacity to appropriately detect diseased patients. It is the same as recall.

#### Specificity

It is a test's ability to correctly identify healthy individuals.

$$Specificity = \frac{TN}{{TN + FP}}$$

#### Receiver operator characteristic (ROC)

This curve displays the variations of sensitivity with respect to a (1-specificity). It is used to demonstrate the relationship between sensitivity and specificity.

#### Area under curve (AUC)

It indicates how successfully the model can differentiate between positive and negative categories.

### Model's training

For model training, forty iterations of the Adam optimizer were utilized. Smaller batch sizes are chosen since they improve the model's test accuracy and expedite the network's capacity to learn. Adam's optimization has a 0.001 percent learning rate. Adam is utilized for training the model since it updates the network weight repeatedly based on the training dataset. The results of adaptive moment estimation in Adam. The dataset is separated into sections for training, validation, and testing. The CXR dataset's validation loss is the condition for epoch termination. The training accuracy is higher than the validation accuracy because the validation data points are newly inserted unseen data points and it gives a general idea how the proposed model will predict unseen samples.

### Performance evaluation of the proposed methodology

In our experiment, we evaluated the performance of Pneumonia prediction on two architectures: (i) QCNN without Attention blocks and (ii) QCNN with spatial and channel attention blocks. The same set of hyper-parameters values as in Table 4 and the dataset in Table 2 has been used to make a comparative analysis. Table 5 presents the performance of both architectures. As in Table 5, we observed a rise of 4% in classification accuracy when attention modules are augmented in the QCNN architecture.

**Table 5:** Performance Comparison between the architectures.

Architecture	Epoch	Total parameters	Trainable parameters	Test accuracy	Precision	Recall	F1-score	AUC	Cohens kappa
Quaternion residual network	40	569,345	560,769	90.27	91.44	95.53	93.44	0.86	0.75
Quaternion residual attention network	40	569,345	560,769	94.53	93.56	98.86	96.14	0.89	0.82

## Analysis of result

**Figure 7:**
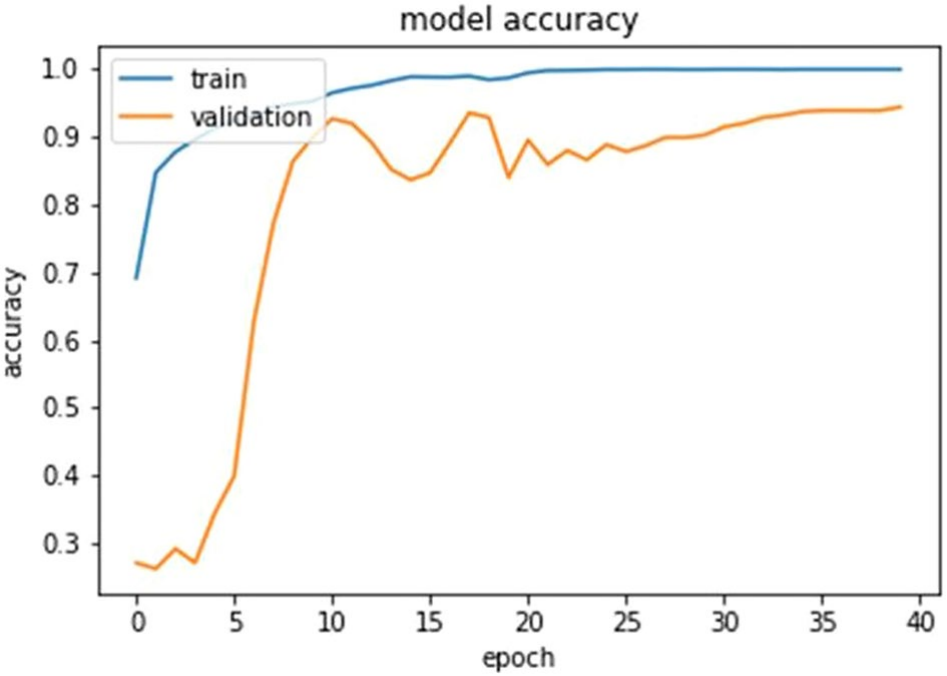
Accuracy curve.

**Figure 8:**
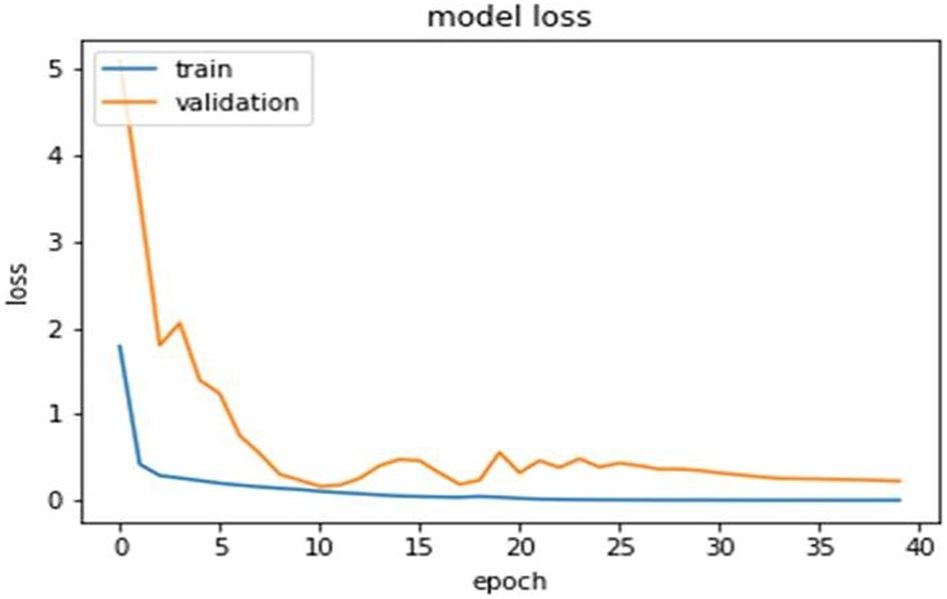
Loss curve.

**Figure 9:**
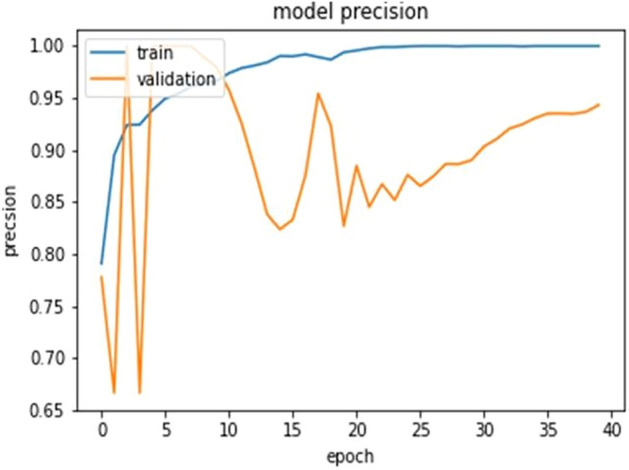
Precision curve.

**Figure 10:**
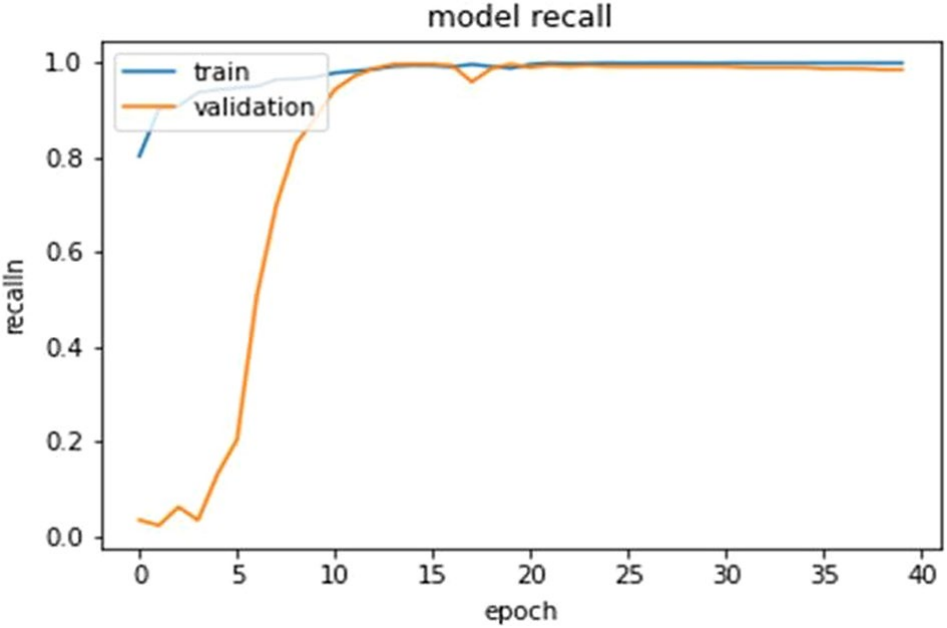
Recall curve.

**Figure 11:**
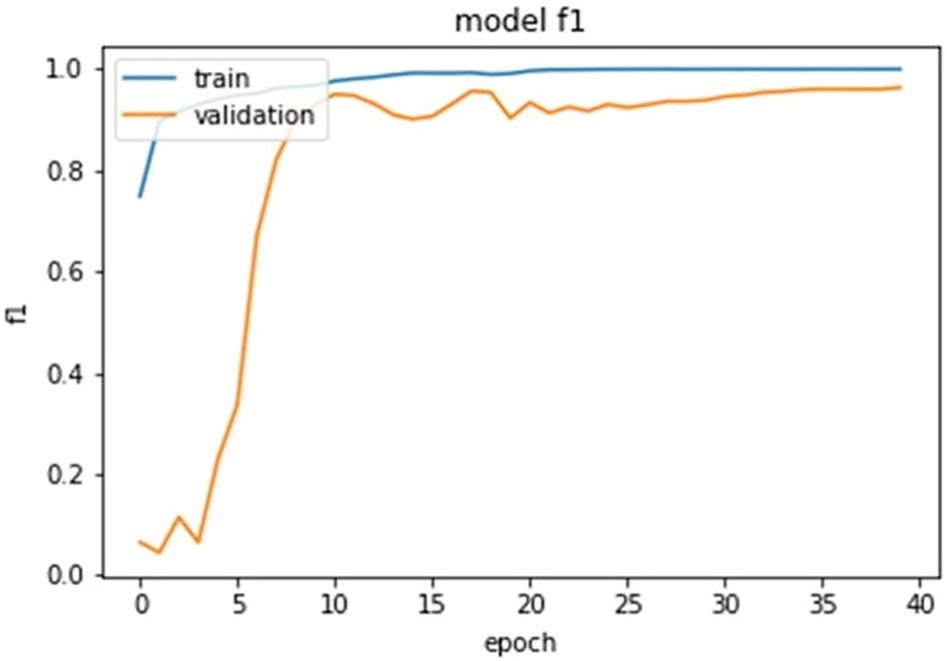
F-1 score curve.

**Figure 12:**
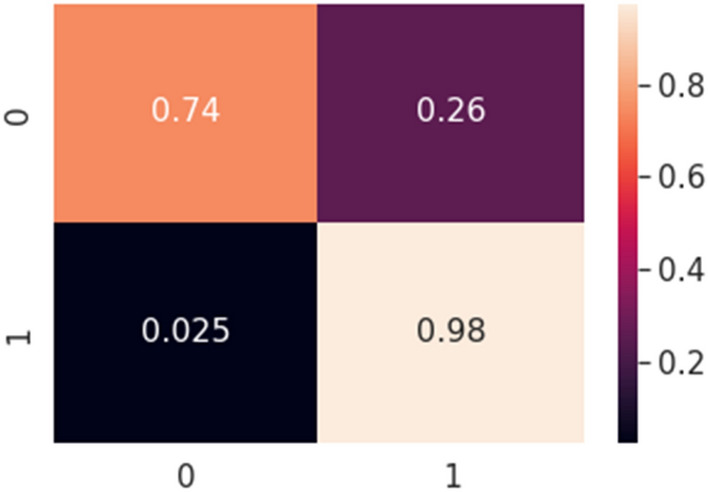
Confusion matrix.

**Figure 13:**
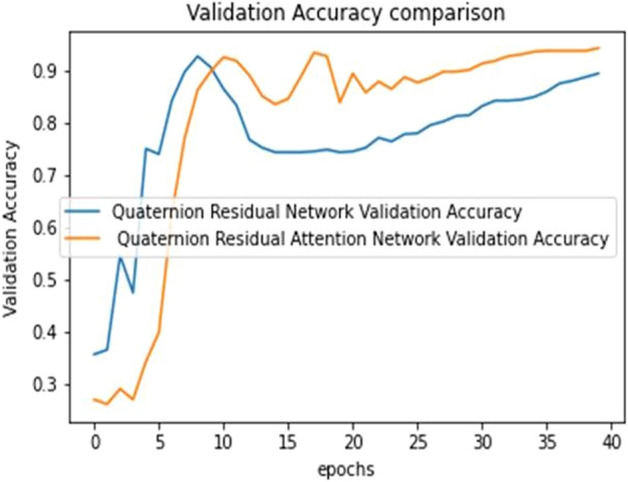
Validation accuracy plot.

**Figure 14:**
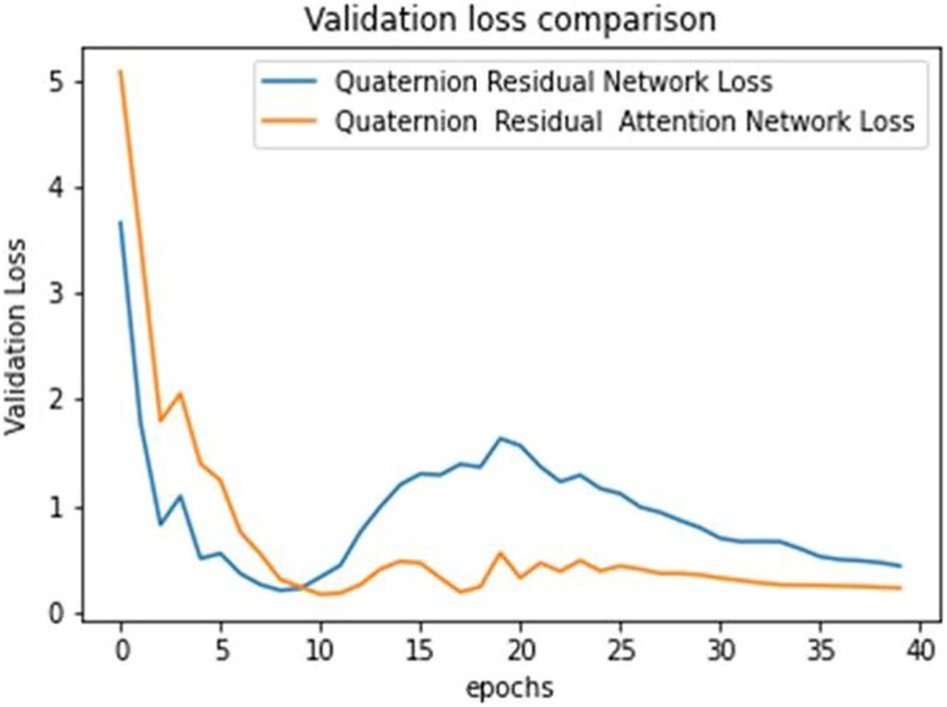
Validation loss plot.

**Figure 15:**
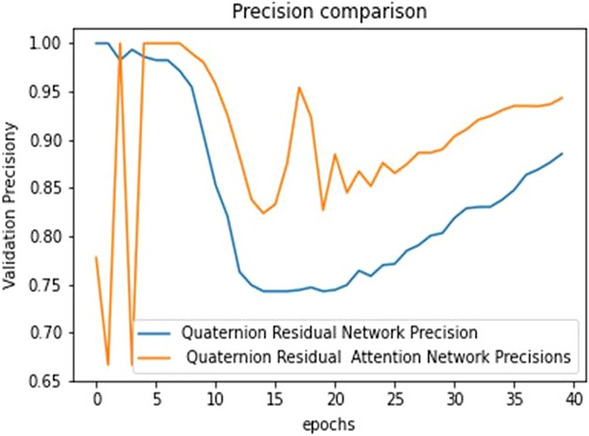
Precision plot.

**Figure 16:**
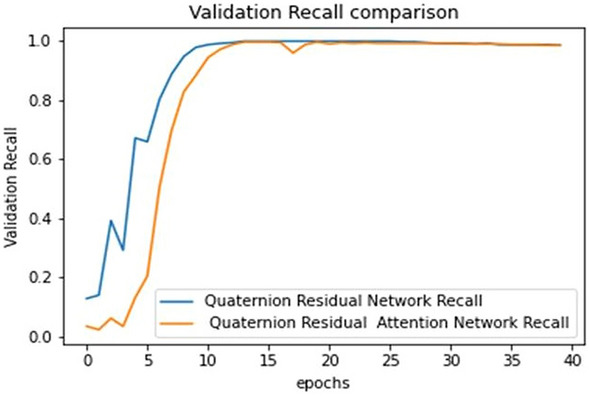
Recall plot.

**Figure 17:**
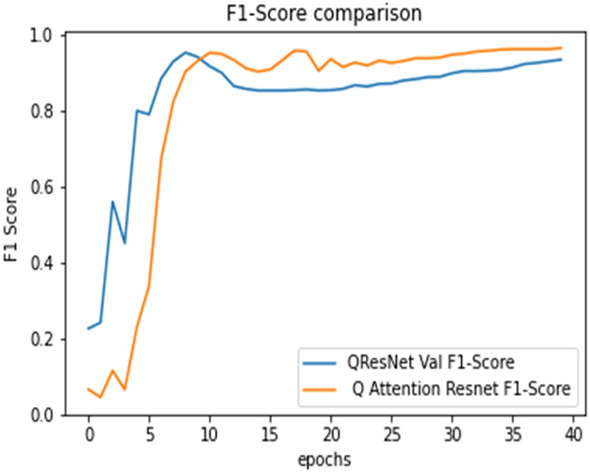
F1-score plot.

The ultimate goal of Pneumonia detection using deep learning is to minimize false positive and negative cases, as they can have significant consequences for patient care. False positives can lead to unnecessary treatments, which can be costly and potentially harmful to the patient, while false negatives can result in delayed diagnosis and treatment, which can be life-threatening. Therefore, in the context of pneumonia detection, it is more important to prioritize accuracy over training and prediction time. Table 5 and Figs. 7, 8, 9, 10, 11 and 12 present the performance of QCNN with spatial and channel attention modules. The performance curve shows the promising results of Pneumonia prediction. Table 5 also shows that there is a significant rise in all performance metrics when spatial and channel attention modules are augmented in QCNN architecture. Figures 13, 14, 15, 16 and 17 show the performance metrics comparison between Pneumonia detection with QCNN and QCNN with attention modules. These Figs. 13, 14, 15, 16 and 17 show that by augmenting the attention mechanism in QCNN, we get a significant rise in performance which improves the result of Pneumonia detection.

**Figure 18:**
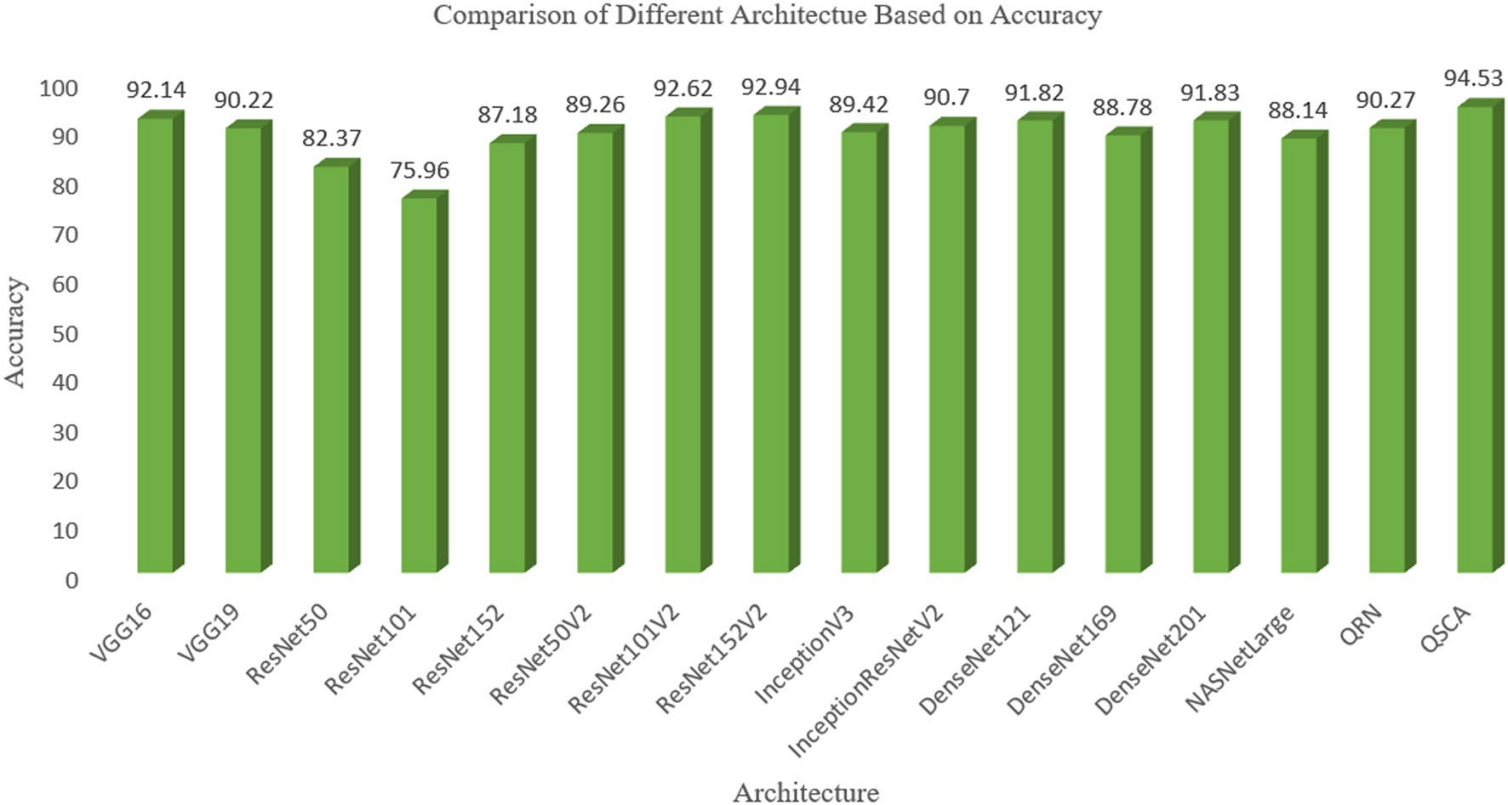
Comparison of accuracy of different deep models at pneumonia dataset.

Whave performed the experiment with this dataset applying different deep learning architectures which is presented by Table 6 with performance metrics such as accuracy, f1-score, number of trainable parameters, and non-trainable parameters. We have presented the accuracy of models by bar graph in Fig. 18, which shows that the proposed method performs better while capturing the complex features and attending the important region of an image.

**Table 6:** Performance comparison with other architectures on the same dataset.

S. no	Architecture	Accuracy	F-score	No of trainable parameter	No of non trainable parameter
1	*VGG*16	92.14	0.9234	50,178	14,714,688
2	*VGG*19	90.22	0.8999	50,178	20,0244,384
3	*ResNet*50	82.37	0.8281	200,706	23,587,712
4	*ResNet*101	75.96	0.7593	200,706	42,658,176
5	*ResNet*152	87.18	0.8734	200,706	58,370,944
6	*ResNet*50*V*2	89.26	0.8937	200,706	23,564,800
7	*ResNet*101*V*2	92.62	0.9250	200,706	42,626,560
8	*ResNet*152*V*2	92.94	0.9312	200,706	58,331,648
9	*InceptionV*3	89.42	0.8937	102,402	21,802,784
10	*InceptionResNetV*2	90.70	0.8989	200,706	58,331,648
11	*DesnseNet*121	91.82	0.9171	100,354	7,037,504
12	*DesnseNet*169	88.78	0.8874	163,074	12,642,880
13	*DesnseNet*201	91.83	0.9171	188,162	18,321,984
14	*NASNetLarge*	88.14	0.8812	975,746	84,916,818
15	*Quaternion residual network*	90.27	0.9344	560,769	8576
16	*QSCA* (*Proposed paper*)	94.53	0.9614	85,800,194	0

## Conclusion and future work

In this research, we provide a system in which deep learning architectures are adapted to the quaternion domain, and it is augmented with attention modules that consist of channel attention and spatial attention modules to focus only on more relevant portions of the image. Quaternion-customized deep neural network architecture shows better classification performance, especially of multi-channel data, because of the real-valued conventional DNN they handle. This architecture was evaluated on a public dataset on Kaggle of CXR images for the detection of pneumonia. We customized the residual network in the quaternion domain. We first evaluated the residual quaternion network on the dataset, and it gave a test accuracy of 90.27%, which is better than real-valued residual CNN architecture. We evaluated quaternion residual network architecture augmented with spatial and channel attention modules, which gave an accuracy of 94.53%. We observed a 4% rise in accuracy in the experiment when the attention mechanism is integrated with Quaternion residual network. The proposed model displays generalization potential when evaluated on distinct data sets. If the proposed architecture is ensembled with predictions of experienced radiologists, it is expected to offer outperforming results, which are left as the future scope of the proposed work.

## Data Availability

The model, data and scripts are all available at https://github.com/singhsukhendra/MyExperiments2023/blob/main/QCSA_for_Pneumonia_Detection.ipynb.

## Abbreviations

CNN: Convolution neural networks
QNN: Quaternion neural networks
QCNN: Quaternion convolution neural networks
CXR: Chest X-ray
RNN: Recurrent neural networks
QCSA: Quaternion channel spatial attention network
